# Conservative management of an infected laparoscopic hernia mesh: A case study^☆^

**DOI:** 10.1016/j.ijscr.2013.08.008

**Published:** 2013-08-27

**Authors:** Duncan Alston, Stephanie Parnell, Bhupinder Hoonjan, Arun Sebastian, Adam Howard

**Affiliations:** Department of Vascular Surgery, Colchester General Hospital, Turner Road, Colchester CO4 5JL, United Kingdom

**Keywords:** Mesh, Infection, Hernia, Laparoscopic, Irrigation

## Abstract

**INTRODUCTION:**

A dreaded complication of laparoscopic hernia repair is infection of the mesh. Traditionally mesh infection is managed by surgical removal of the mesh, an extensive procedure resulting in high re-herniation rates. A technique to treat such infections whilst salvaging the mesh is sorely needed. We describe a case in which a laparoscopic mesh infection was treated solely with drainage, parenteral antibiotics and antibiotic irrigation of the mesh.

**PRESENTATION OF CASE:**

A 65 year old gentleman presented 11 months post laparoscopic repair of an inguinal hernia with malaise and an uncomfortable groin swelling. Computed tomography scanning revealed a collection surrounding the mesh which was drained and cultured to show heavy growth of Staphylococcus aureus. A pigtail drain on continuous drainage was inserted and kept in situ for 7 weeks.

The patient received one week of intravenous flucloxacillin and two gentamycin irrigations through the drain as an inpatient. He then received 6 weeks of oral flucloxacillin and bi-weekly saline flushes through the drain in the community. By 12 weeks an ultrasound scan showed resolution of the collection. At 7 months he remains clinically free from recurrence.

**DISCUSSION:**

Here we report a novel conservative method used to treat a hernia mesh infection, preserve the mesh and avoid major surgery. Other reports exist suggesting variations in conservative methods to treat mesh infections, however ours is by far the most conservative.

**CONCLUSION:**

Clearly, further research is required to identify which method is most effective and in which patients it is likely to be successful.

## Introduction

Laparoscopic hernia repair is an increasingly widely used technique. It has a number of advantages over open hernia repair. These include a reduced operating time and length of patient stay, reduction in post-operative infection and abscess formation as well as showing trends towards a reduction in hernia recurrence.^1^ Modern hernia repairs almost always use a prosthetic mesh material as the ‘tension-free’ repair they provide has been shown to significantly reduce hernia recurrence.^2^

A severe complication of laparoscopic hernia repair is infection of the mesh. Patients who smoke or who have recurrent hernias are more likely to develop mesh infection,^3^ but the increased risk of recurrence without using a mesh often outweighs the risk of infection in these patients.

Mesh infections have traditionally been treated with large reconstructive operations to remove the mesh, often leaving the patient with high re-herniation rates. Conservative methods to treat this complication whilst also preserving the mesh are needed. Here we present a laparoscopic mesh infection successfully treated conservatively with oral antibiotics and a pig-tail drain for drainage and gentamycin irrigation; a technique only reported twice before in the literature in this context.^4,5^

## Presentation of case

A 65 year old gentleman was seen 11 months after a laparoscopic repair of a left inguinal hernia. His hernia had been repaired with a Parietex Tyco mesh. At the time of the operation he had received 1.2 g of co-amoxyclav (1 g of amoxicillin and 200 mg of clavulinic acid) 30 min prior to incision. Until the presentation described below the post-operative course had been unremarkable (the patient experienced no pain or fever) and consequently no post-operative imaging was performed.

Eleven months post-operatively he was seen in clinic complaining of a 3 week history of general malaise, raised temperatures and an uncomfortable swelling in his left groin. On examination the swelling was mildly tender, fluctuant, and dull to percussion. There was erythema and warmth over the left iliac fossa.

**Fig. 1:**
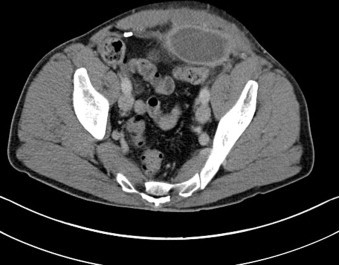
Computed tomography image showing a left sided collection at the site of a laparoscopically inserted hernia repair mesh.

A computed tomography scan of the patient's abdomen demonstrated a collection of pus around the intra-abdominal hernia mesh (Fig. 1) which was then aspirated under ultrasound guidance. The pus cultured a heavy growth of *Staphloccocus aureus* which was sensitive to gentamicin, flucloxacillin, trimethoprim, ciprofloxacin and clarithromycin but resistant to penicillin.

The patient was then admitted and a pigtail drain was inserted and left on continuous drainage. Treatment was started with intravenous (IV) flucloxacillin 1gram four times per day for 7 days and Gentamicin 250milligrams once daily for 2 days.

A further culture was taken from the drain three days after insertion which again grew “*Stapholococcus aureus*+/−” with the same sensitivities.

By the 6th day after drain insertion the pus drainage had become minimal (<20 ml/day) and a gentamicin flush was performed. 120 mg of gentamicin in 20 ml of normal saline was pushed through the drain into the cavity around the mesh and left for 30 min with the drain clamped. The drain was then returned to free drainage. This was then repeated on the 8th day after insertion.

The patient was then discharged with 12 weeks of oral flucloxacillin 500 mg 4 times a day and 6 weeks of saline flushes were performed (using an aseptic technique) in the community twice weekly by the district nurses. The drain eventually fell out after seven weeks.

**Fig. 2:**
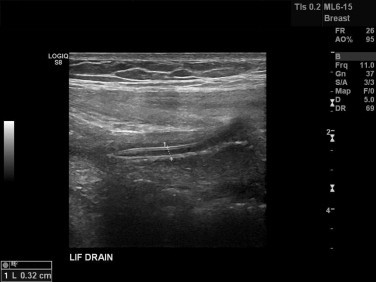
Ultrasound image showing resolution of the collection (1) around the laparoscopically inserted hernia repair mesh.

By twelve weeks post discharge there was complete resolution of his initial signs and symptoms and an ultrasound scan (Fig. 2) and microbiology culture confirmed the absence of a recurrent infection. At 7 months post-admission he remains clinically free from recurrence.

## Discussion

Here we present a case of a laparoscopic hernia mesh infection being successfully treated conservatively with drainage and parenteral and local antibiotic treatment. This avoided the need for considerable reconstructive surgery.

A number of techniques employed peri-operatively are used to try to prevent infection. A recent Cochrane meta-analysis of 9 randomised controlled trials found antibiotic prophylaxis at the time of surgery to significantly reduce infections in open hernia repairs^6^ (odds ratio 0.61, 95% confidence interval 0.4–0.92). Experimental studies in rats have also shown significant decreases in MRSA growth on meshes soaked in vancomycin before implantation.^7^ However, despite good evidence suggesting methods to prevent graft infection, evidence assessing how to deal with infection is mostly anecdotal.

Aguilar et al.^4^ describe 3 cases of infected ventral hernia meshes. These patients were treated with three times daily gentamycin irrigation through local drains and one month of IV antibiotics. Trunzo et al.^5^ describe 2 cases of laparoscopic ventral hernia mesh repair infection. Similarly, both were treated with 4 weeks of three times daily gentamycin irrigations. One case was treated with 2 weeks of IV vancomycin and the other with ciprofloxacin. Both of these papers report good outcomes. Interestingly, our patient demonstrated similar outcomes but with only 2 gentamycin irrigations and only 1 week of IV antibiotics, followed by 6 weeks of oral antibiotics and bi-weekly saline flushes. Consequently our patient had a much shorter hospital stay than the other patients described here and had a greatly reduced risk of gentamycin toxicity from long term use.

Variations on this technique are also described in the literature. More aggressive methods of infected mesh salvage are reported by Paton et al.^8^ who reports successful mesh salvage after wound debridement, partial mesh removal, vacuum dressing application and IV antibiotics. Similarly a recent case series by Meagher et al.^9^ show success in treating infected meshes using local wound care, vacuum dressings and antibiotics only if clinically indicated. Finally, Ruiz-Tovar et al.^10^ successfully achieved resolution of mesh infection by re-opening the wound, treating with local pressurised gentamycin irrigation three times a day and IV co-amoxyclav for 7 days.

As can be seen, considerable variation exists in the techniques used to conservatively treat hernia mesh infections. More research is clearly needed to determine whether, for example, wound opening and debridement is needed or whether a drain is sufficient. Furthermore, varying course durations of gentamycin irrigation and intravenous antibiotic treatment clearly affect the length of time a patient must remain in hospital and have considerations for antimicrobial stewardship. These are issues that need to be addressed by further research before conservative treatment of hernia mesh infections can become a widely used technique.

A final issue exists. Most of the current evidence discussed above is from case reports. Cases where conservative management of hernia mesh infection is attempted but fails are not being reported. Hence, prospective trials need to be conducted to look at the rate of success and to help decide in which patients conservative management is more likely to be successful.

## Conclusion

In conclusion, we have described a conservative method to treat a patient who was clinically unwell with a laparoscopic hernia mesh infection. The method we describe is one of the most conservatives in the literature: our patient was only admitted for 1 week, only had 2 irrigations with gentamycin and did not require any debridement or surgery. However, it is clear that further research is warranted to help decide which patients to select for conservative management of mesh infections and which regimen is best.

## Conflict of interest

The authors declare no conflict of interest.

## Funding

None.

## Consent

Written informed consent was obtained from the patient for publication of this case report and accompanying images. A copy of the written consent is available for review by the Editor-in-Chief of this journal on request.

## Author contributions

Duncan Alston – literature review, writing the paper.

Stephanie Parnell – writing the ‘Case Report’ part of the paper.

Bhupinder Hoonjan – preparation of the images.

Arun Sebastian – reporting images.

Adam Howard – final approval of submitted manuscript.
